# Optimization of Concentration-Time, Agar, and Sugar Concentration for Sweet Gelatinized Adzuki-Bean Jelly Cake (Yokan) by Response Surface Methodology

**DOI:** 10.3390/gels8090540

**Published:** 2022-08-27

**Authors:** Ping-Hsiu Huang, Yu-Tsung Cheng, Wen-Chien Lu, Po-Hsien Li

**Affiliations:** 1 School of Food, Jiangsu Food and Pharmaceutical Science College, Huai’an 223003, China; 2 Cardiovascular Center, Taichung Veterans General Hospital, Taichung 40705, Taiwan; 3 Department of Food and Beverage Management, Chung-Jen Junior College of Nursing, Health Sciences and Management, Chia-Yi City 60077, Taiwan; 4 Department of Food and Nutrition, Providence University, Taichung 43301, Taiwan

**Keywords:** yokan, sweet gelatinized adzuki-bean jelly cake, response surface methodology, gelatin, quality

## Abstract

Samples of sweet gelatinized adzuki-bean jelly cake were successfully prepared and systematically analyzed to investigate the factors that affect the production, quality, and gelatin properties of yokan (gelatinized adzuki bean cake). The purpose of this study was to investigate the properties of gelatinized adzuki-bean cake gelatin and identify the optimal production conditions using response surface methodology with three factors: agar concentration, sugar concentration, and concentration time. Findings show that the optimum processing conditions are 1.2–1.5% agar concentration and 34–40% sugar concentration, with 30–40 min concentration time. These conditions produced a gelatinized adzuki-bean cake favored by the majority of the sensory evaluators. Overall, the relationships between different gelatinized adzuki-bean cake processing conditions and gelatin properties were preliminarily clarified. The findings not only provide a promising avenue for gelatinized adzuki-bean cake production but also promote the potential application of various processing conditions in quality improvement.

## 1. Introduction

Adzuki beans (*Vigna angularis*) have been traditionally utilized in Chinese herbal medicine and primarily in Asia as a filling in cuisine, snack foods, or candy products for thousands of years [1]. It is one of the popular legumes [2] because of its wealth of starch, digestible proteins, elements of minerals, and vitamins. The most common bean processing methods include splitting (peeling), soaking, sprouting, boiling, and pressure cooking [3]. As a snack food or candy, yokan (gelatinized adzuki-bean cake) is primarily produced from adzuki-bean paste, sugar, and agar, which are heat-cooked [4].

Yokan was introduced to Japan from China by monks who learned Buddhist teachings during the Tang Dynasty. Initially, it was made by cooking the gelatin from lamb derived from lamb soup and lamb liver soup. When the soup cools, the gelatin in the soup coagulates into a jelly-like texture [5]. In Japan, yokan was continuously improved by making it with agar or other gelatinizing agents, sugar, and azuki-bean paste to form a homogeneous gelatinous state. It became a famous dessert that is used during tea ceremonies in Japan [6]. Presently, it is a sweet dessert renowned throughout Asia. However, food cultures differ between countries or regions. Yokan is culturally familiar to Asia but unfamiliar to Westerners. Groups with various food cultures likely have different levels of cultural familiarity with their particular ethnic foods [7]. The adhesiveness and dispersibility of gelatinized adzuki-bean cake play an important role in product quality. However, each food has a different chemical composition and material structure, which will show various characteristics. Gelatinized adzuki-bean cake is a jelly-like gelatinous solid whose gelatinous properties were obtained with agar, a polysaccharide extracted from red algae, and is thermally reversible. The most well-known gelling mechanism is the domain model proposed by Rochas, C. and M. Rinaudo [8]. In the hot solution state (above the gelling temperature), the molecular structure forms coils and then changes to a regular double helix at lower temperatures. The double helix continues to aggregate forming adhesive regions. The whole system forms a gelling network structure. Wongphan, P. and N. Harnkarnsujarit [9] proposed that the interaction between starch and agar caused poor water solubility due to the same molecular binding, while the agar concentration (10–30%) showed no significant effect. In addition to adzuki-bean paste as the primary ingredient of gelatinized adzuki-bean cake, the structural completeness, and the interaction with sugar and agar is important. It affects the texture characteristics, the syneresis (%) of gelatinized adzuki-bean cake, and sensory evaluation preferences. Phase changes influence the internal molecular structure of food gel, altering the various forces within the polymer, including hydration-ion pressure, rubber elasticity, and polymer–polymer affinity, Their sum determines whether the gel will absorb or exclude the osmotic pressure of the liquid within the gel [10]. 

A survey found that consumers generally have high acceptability of gelatinized adzuki-bean cake products with fineness, the aroma of beans, smoothness, chewiness, and appropriate sweetness. However, commercially available gelatinized adzuki-bean cake products depend on the recipe and expert experience for monitoring the quality without any scientific basis or index. The study aims to identify the theoretical basis, quality factors, and indexes while understanding the gelling mechanisms, thereby contributing to the processing of bean-paste-related products and innovative product development. Hence, the results provide useful information for developing adzuki-bean-based food sweeteners.

## 2. Materials and Methods

### 2.1. Materials

Adzuki beans (*Vigna angularis* [Willd.] Ohwi et Ohash) were randomly harvested among 10 adzuki bean plants from a local farm in Agricultural Research and Extension Station (Tainan District, Taiwan) and dried at 40 °C in an air-conveyor oven, while the moisture content was controlled between 10.0 ± 0.3 with 8–12 h drying time. Then, stored in vacuum packaging at 5 °C until use. Food-grade agar powder was purchased from Hushing Food Co., Ltd. (Taichung City, Taiwan). Sugar was purchased from Taiwan Sugar Co., Ltd. (Tainan City, Taiwan). All chemical reagents were of analytical grade.

### 2.2. Experimental Design of Response Surface Methodology for the Gelatinized Adzuki-Bean Cake

**Figure 1:**
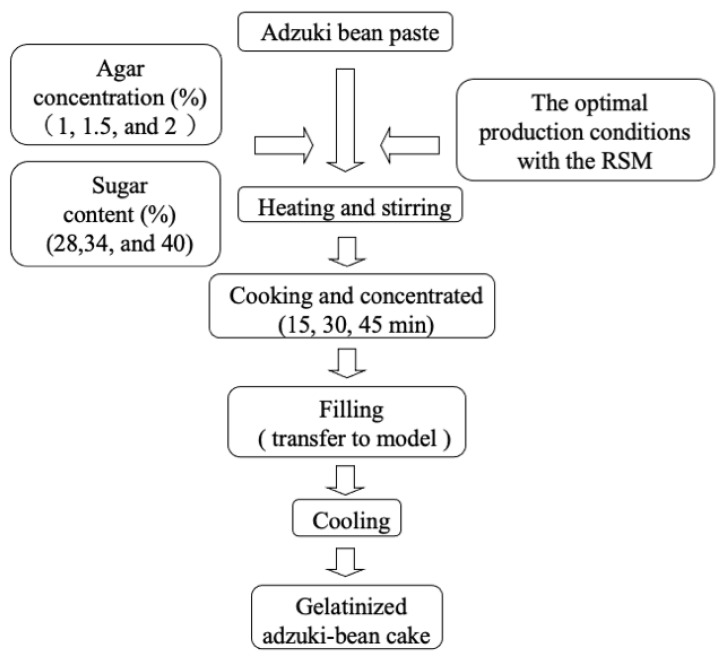
Experimental design of gelatinized adzuki-bean cake.

The study used Design-Expert software version 10 (Statease Inc., Minneapolis, MN, USA) for experimental design with further statistical analysis and regression analysis using response surface methodology (RSM). The experimental design (Figure 1), according to the three variables-three levels responses surface analysis proposed by Box and Behnken [11] was used to investigate the amounts of sugar and agar that should be added to the same filling amount of adzuki-bean paste (Table 1). Additionally, the concentration of agar solution (X) was 1.0%, 1.5%, and 2.0%; the sugar content (Y) was 28%, 34%, and 40%; and the concentration time (Z) was 15, 30, and 45 min (Table 2), which were used as the independent variables. The gelatinized adzuki-bean cake’s characteristics, such as moisture content, color, gelatin strength, hardness, viscosity and elasticity, and sensory evaluation, were used as the independent variables to determine the optimum processing conditions for producing gelatinized adzuki-bean cake. 

### 2.3. Syneresis (%)

The detection method was modified according to the approach proposed by Lin et al. [12] and Wu et al. [13]. Two Whatman No. 1 filter paper (150 mm) strips were used to wrap the sample, and then the sample was placed in a centrifuge tube to be centrifuged at 1000 rpm for 15 min. To determine the release of water from the gelatinized adzuki-bean cake by centrifugation, the water content was divided by the weight of the sample; the resultant percentage was determined as the syneresis (%). 

### 2.4. Chromatic Analysis

The *L*, *a*, and *b*-values of the samples were determined using Color Meter ZE-2000 (Nippon Denshku Industries Co., Ltd., Tokyo, Japan) for each sample in three replicates.

### 2.5. Texture Analysis

Texture analysis was performed with the Sun rheometer CR-200D (Sun Scientific Co., Ltd., Tokyo, Japan) at room temperature (25 ± 2 °C) using an adapter with a 5 mm diameter spherical plunger, with a carrier table rise speed of 200 mm/min and a penetration range of 5 mm, repeated twice. A total of 20 replicates were performed for each sample. The measured items include hardness, viscosity, and elasticity.

### 2.6. Sensory Evaluation

A nine-point scale was used to evaluate the preference, with a scale of 1 meaning “extremely dislike”, 5 meaning “neither like nor dislike”, and 9 meaning “extremely like”, while each evaluated four parts: appearance, flavor, texture, and overall acceptability. The evaluation panel comprised 30 participants who had received training as sensory tasters.

### 2.7. Sugar Content (°Brix) 

The sample of 1.0 g was added to nine-fold water and ground with a mortar for 5 min at room temperature, followed by filtration with Whatman No. 4 filter paper to remove solids. The filtrate was measured with an N-1E Hand refractometer (ATAGO Co., Ltd., Tokyo, Japan) and was expressed as °brix.

### 2.8. Microphotographic Observations

The microstructure was observed under a penetrating optical microscope (Eclipse E400, Nikon, Co., Ltd., Tokyo, Japan) with a 1:1 mixture of glycerol and water formulated at a 3% concentration with different adzuki bean samples. Gelatinized adzuki-bean cake samples (34% sugar content and commercially available products) were soaked in 10% formalin to fix the tissues, while structurally fixed samples were dehydrated using an autotechnicon (Shandon Citade 1000, GE Healthcare, Boston, MA, USA), followed by wax embedding (paraffin-embedded Shandon HispoCenter 2, GE Healthcare, Boston, MA, USA). The embedded waxes were sectioned by a slicer (Microtome, Shandon AS325, GE Healthcare, Boston, MA, USA) at a thickness of 4-μm, dewaxed with xylene, stained with hematoxylin and eosin for protein staining, dewatered, sealed, and then observed under an optical microscope (E400, Nikon Co., Ltd., Tokyo, Japan) to determine the microstructure of the adzuki beans.

### 2.9. Scanning Electron Microscope Micrographs

The freeze-dried gelatinized azuki-bean cake samples, which included 34% sugar content, sugar-free and commercially available products, were adhered to an aluminum stab (Topon Co., Ltd., Tokyo, Japan) with double-sided adhesive and then coated with gold coating under vacuum for 90 s with an ion sputter coater (JBS-ES 150 model, Topon Co., Ltd., Tokyo, Japan), followed by scanning electron microscopy (SEM) (ABT-150S, Topon Co., Ltd., Tokyo, Japan) for observation and photography. 

### 2.10. Statistics Analysis

Data were analyzed using one-way ANOVA and Duncan’s multiple range test using IBM SPSS Statistics version 18.0 (International Business Machines Corporation, Armonk, NewYork, NY, USA). The criterion for significance was set at *p* = 0.05. All the data were presented as mean ± standard deviation (SD). The results of the response surface model experiment were analyzed using the SAS software application and the RSREG program, and the resultant multinomial plots were regressed using the Surfer access system (Version 3.00, Golden Software Inc., Golden, CO, USA).

## 3. Results and Discussion

### 3.1. RSM

In this study, the most critical factors affecting the quality of gelatinized adzuki-bean cake were identified based on the results of the preliminary tests, which included the quality and content of bean paste, the sugar content, the agar concentration, and the concentration time, which were investigated to determine the effects of these factors (independent variables) on the production of gelatinized adzuki-bean cake with the same bean paste ingredients (self-made sugar-free bean paste). In the case of the gelatinized adzuki-bean cake, measured by physicochemical methods with the combination of various processing conditions, data on the different response variables of gelatinized adzuki-bean cake were obtained (Table 3). Afterward, Response Surface Regression (RSREG) parameters in SAS were used to conduct statistical regression analysis based on the analytical results of SAS [14]. The analysis of variance (ANOVA) results for each variable and the processing independent variable, and the results of the sum effect of the processing independent variable (Table 4 and Table 5) were obtained as follows. The variability of gelatinized adzuki-bean cake in terms of *b*-value, fineness, aroma, and overall acceptability was 68.97, 52.97, 69.92, and 71.85, respectively, lower than the variability required for the regression analysis (R^2^ > 80%). The linear, quadratic, and cross-product in Table 4 are nonsignificant. The agar concentration, sugar content, and concentration time showed negative effects on the *b*-value, fineness, aroma, and overall acceptability of gelatinized adzuki-bean cake; thus, these three factors were not the primary factors affecting these four properties [15,16], resulting in a low correlation with the regression patterns obtained from the study. Additionally, according to the syneresis (%), *L*, *a*-values, texture analysis (including gelatin strength, hardness, viscosity, and elasticity), and sensory evaluation (color, softness, chewiness, and granularity preference) of gelatinized adzuki-bean cake, the variability of the results exceeded 80%, thus matching the required variability in regression analysis (R^2^ > 80%) [17].

The significance of linear, quadratic, and cross products in Table 4 indicates that the three factors of agar concentration, sugar content, and concentration time adopted in this experiment influenced the syneresis, chromatic parameters (brightness and redness), texture (including gel strength, hardness, stickiness, and elasticity), and sensory evaluation of gelatinized adzuki-bean cake. Hence, the results are highly correlated with the obtained regression patterns. In the course of analyzing the data, residuals were examined for lack of fit when comparing the variability. Generally, the lack-of-fit of the model was significant (*p* < 0.05), which means that the surface model created by the quadratic polynomial was not appropriate to describe the variation of the physical-chemical properties of samples. The above ANOVA of process factors indicates that the regression patterns of the syneresis, chromatic parameters, texture, and sensory evaluation of gelatinized adzuki-bean cake were statistically insignificant (*p* > 0.05) with high variability, which means that the patterns are statistically significant and correlate with each other. These findings can be used to describe the various physicochemical properties of the samples. The variance analysis table shows that the process variables of the study correlated well with the independent variables of the gelatinized adzuki-bean cake production. The quality of the gelatinized adzuki-bean cake can be explained based on the RSM by multiple equations. For each response variable, the regression coefficients were obtained, and the following three-factor quadratic polynomial was formulated according to different response properties:

Syneresis (%) = 14.17 − 4.39X + 0.83Y − 5.14Z − 0.57X^2^ − 1.03XY − 1.69Y^2^ + 2.58XZ + 0.63YZ − 1.33Z^2^ (1)

*L*-value = 11.10 + 0.62X − 1.03Y − 0.13Z + 0.47X^2^ − 0.08XY + 1.03Y^2^ + 0.74XZ + 0.21YZ − 0.22Z^2^ (2)

*a*-value = 8.89 − 0.07X − 0.40Y − 0.49Z − 0.32X^2^ − 0.12XY − 0.35Y^2^ + 0.08XZ + 0.05YZ − 0.39Z^2^ (3)

Gel strength = 23.00 + 6.03X − 2.69Y + 8.70Z + 1.27X^2^ − 1.45XY + 1.68Y^2^ − 2.82XZ − 2.11YZ + 4.97Z^2^ (4)

Hardness = 0.5753 + 0.2098X − 0.0205Y + 0.2170Z + 0.0012X^2^ + 0.0411XY + 0.0149Y^2^ + 0.0297XZ − 0.0377YZ + 0.0918Z^2^ (5)

Viscosity = 2,080,592 + 777,387X + 4050.17Y + 640,577Z − 157,119X^2^ + 239,779XY − 35,083Y^2^ + 269,696XZ + 53,906YZ + 66,430Z^2^ (6)

Elasticity = 3,582,642 + 1,248,486X − 126,251Y + 1,265,754Z − 54,027X^2^ + 222,187XY + 72,637Y^2^ + 193,174XZ − 122,002YZ + 497662Z^2^ (7)

Sensory evaluation of color preference = 5.88 + 0.16X − 0.12Y + 0.35Z + 0.25X^2^ + 0.38XY − 0.06Y^2^ − 0.19XZ − 0.19YZ + 0.12Z^2^ (8)

Sensory evaluation of hardness and softness preference = 6.34 − 0.25X + 0.19Y + 0.03Z − 0.42X^2^ + 0.06XY − 0.17Y^2^ + 0.06XZ − 0.19YZ − 0.67Z^2^ (9)

Sensory evaluation of chewing preference = 5.78 + 0.0013X + 0.30Y + 0.11Z − 0.29X^2^ − 0.16XY − 0.26Y^2^ − 0.03XZ − 0.31YZ − 0.20Z^2^ (10)

Sensory evaluation of granularity preferences = 6.30 − 0.19X + 0.16Y + 0.06Z − 0.24X^2^ + 0.13XY − 0.37Y^2^ + 0.19XZ − 0.31YZ + 0.24Z^2^ (11)

This study aimed to investigate the properties of gelatinized adzuki-bean cake gelatin and the optimum production conditions, including three factors: agar concentration, sugar content, and concentration time. Therefore, while discussing the physicochemical properties of the processed gelatinized adzuki-bean cake, the significantly correlated factors were selected as the variables to be analyzed. In other words, the factors with less significant effects were identified as the control variables, whereas the factors with significant positive effects were considered for discussion. The results of the various responses of the process variables by the independent variables are shown in Table 5. For the syneresis of gelatinized adzuki-bean cake, the concentration time and agar concentration had more influence. Thus, the sugar content was fixed, while the agar concentration was used as the cross coordinate with concentration time as the vertical coordinate to investigate the relationship of the variables. Following the analysis results in Table 5, the RSM equations for each physicochemical property under different conditions (conditions shown in parentheses) were obtained by fixing different independent variables according to different response variables and were substituted into the original quadratic equation with −1, 0, and 1, respectively. Hence, the RSM equations for each physicochemical property under different conditions (conditions in parentheses) are as follows:

Syneresis_1_ (%) = 11.6 5 − 3.36X − 5.77Z − 0.57X^2^ + 2.58XZ − 1.33Z^2^ (Sugar content = 28%)(12)

Syneresis2 (%) = 14.17 − 4.39X − 5.14Z − 0.57X^2^ + 2.58XZ − 1.33Z^2^ (Sugar content = 34%)(13)

Syneresis3 (%) =13.31 − 5.42X − 4.51Z − 0.57X^2^ + 2.58XZ − 1.33Z^2^ (Sugar content = 40%) (14)

*L*-value_1_ = 11.01 − 0.12X + 1.24Y + 0.47X^2^ − 0.08XY + 1.03Y^2^ (Concentrating time = 15 min) (15)

*L*-value_2_ = 11.10 + 0.62X − 1.03Y + 0.47X^2^ − 0.08XY + 1.03Y^2^
(Concentrating time = 30 min) (16)

*L*-value_3_ = 10.75 + 1.36X − 0.82Y + 0.47X^2^ − 0.08XY + 1.03Y^2^ (Concentrating time = 45 min) (17)

*a*-value_1_ = 8.64 − 0.57Z − 0.28Y − 0.39Z^2^ + 0.05ZY − 0.35Y^2^ (Agar concentration = 1%) (18)

*a*-value_2_ = 8.89 − 0.49Z − 0.40Y − 0.39Z^2^ + 0.05ZY − 0.35Y^2^ (Agar concentration = 1.5%) (19)

*a*-value_3_ = 8.50 − 0.41Z − 0.50Y − 0.39Z^2^ + 0.05ZY − 0.35Y^2^ (Agar concentration = 2%) (20)

Gel strength_1_ = 27.37 + 7.48X + 10.81Z + 1.27X^2^ − 2.82XZ + 4.97Z^2^ (Sugar content = 28%) (21)

Gel strength_2_ = 23.00 + 6.03X + 8.70Z + 1.27X^2^ − 2.82XZ + 4.97Z^2^ (Sugar content = 34%) (22)

Gel strength_3_ = 21.99 + 4.58X + 6.59Z + 1.27X^2^ − 2.82XZ + 4.97Z^2^ (Sugar content = 40%) (23)

Hardness_1_ = 0.6107 + 0.1687X + 0.2547Z + 0.0012X^2^ + 0.0297XZ + 0.0918Z^2^ (Sugar content = 28%) (24)

Hardness_2_ = 0.5753 + 0.2098X + 0.2170Z + 0.0012X^2^ + 0.0297XZ + 0.0918Z^2^ (Sugar content = 34%) (25)

Hardness_3_ = 0.5697 + 0.2508X + 0.1793Z + 0.0012X^2^ + 0.0297XZ + 0.0918Z^2^ (Sugar content = 40%) (26)

Viscosity_1_ = 2041458.9 + 537608X + 586617Z − 157119X^2^ + 269696XZ + 66430Z^2^ (Sugar content = 28%) (27)

Viscosity_2_ = 2,080,592 + 777387X + 640577Z − 157119X^2^ + 269696XZ + 66430Z^2^ (Sugar content = 34%) (28)

Viscosity_3_ = 2049559.10 + 1017166X + 69448Z − 157119X^2^ + 269696XZ + 66430Z^2^ (Sugar content = 40%) (29)

Elasticity_1_ = 3781530 + 1026299X + 1387756Z − 54027X^2^ + 193174XZ + 497662Z^2^ (Sugar content = 28%) (30)

Elasticity_2_ = 3,582,642 + 1248486X + 1265754Z − 54027X^2^ + 193174XZ + 497662Z^2^ (Sugar content = 34%) (31)

Elasticity_3_ = 3,529,028 + 1470673X + 1143752Z − 54027X^2^ + 193174XZ + 497662Z^2^ (Sugar content = 40%) (32)

Sensory evaluation of color preference_1_ = 5.94 − 0.22X + 0.54Z + 0.25X^2^ − 0.19XZ + 0.12Z^2^ (Sugar content = 28%) (33)

Sensory evaluation of color preference_2_ = 5.88 + 0.16X + 0.35Z + 0.25X^2^ − 0.19XZ + 0.12Z^2^ (Sugar content = 34%) (34)

Sensory evaluation of color preference_3_ = 5.70 + 0.54X + 0.16Z + 0.25X^2^ − 0.19XZ + 0.12Z^2^ (Sugar content = 40%) (35)

Sensory evaluation of hardness and softness preference_1_ = 5.98 − 0.31X + 0.22Z − 0.42X^2^ + 0.06XZ − 0.67Z^2^
(Sugar content = 28%) (36)

Sensory evaluation of hardness and softness preference_2_ = 6.34 − 0.25X + 0.03Z − 0.42X^2^ + 0.06XZ − 0.67Z^2^ (Sugar content = 34%) (37)

Sensory evaluation of hardness and softness preference_3_ = 6.36 − 0.19X − 0.16Z − 0.42X^2^ + 0.06XZ − 0.67Z^2^ (Sugar content = 40%) (38)

Sensory evaluation of chewing preference_1_ = 5.4887 + 0.14Z + 0.46Y − 0.20Z^2^ − 0.31ZY − 0.26Y^2^ (Agar concentration = 1%) (39)

Sensory evaluation of chewing preference_2_ = 5.78 + 0.11Z + 0.30Y − 0.20Z^2^ − 0.31ZY − 0.26Y^2^ (Agar concentration = 1.5%) (40)

Sensory evaluation of chewing preference_3_ = 5.4913 + 0.08Z + 0.14Y − 0.20Z^2^ − 0.31ZY − 0.26Y^2^ (Agar concentration = 2%) (41)

Sensory evaluation of granularity preferences_1_ = 6.25 − 0.13Z + 0.03Y − 0.24Z^2^ − 0.31ZY − 0.37Y^2^ (Agar concentration = 1%) (42)

Sensory evaluation of granularity preferences_2_ = 6.30 + 0.06Z + 0.16Y − 0.24Z^2^ − 0.31ZY − 0.37Y^2^ (Agar concentration = 1.5%) (43)

Sensory evaluation of granularity preferences_3_ = 5.87 + 0.25Z + 0.29Y − 0.24Z^2^ − 0.31ZY − 0.37Y^2^ (Agar concentration = 2%) (44)

**Figure 2:**
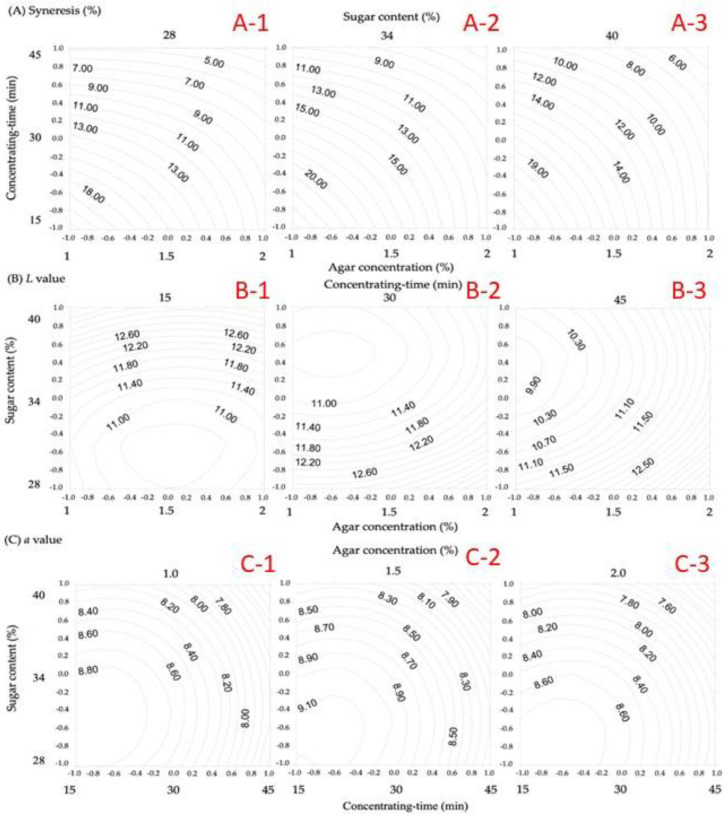
Contour plot of the processing conditions for the gelatinized adzuki-bean cake with respect to syneresis *L* and *a*-value.

**Figure 3:**
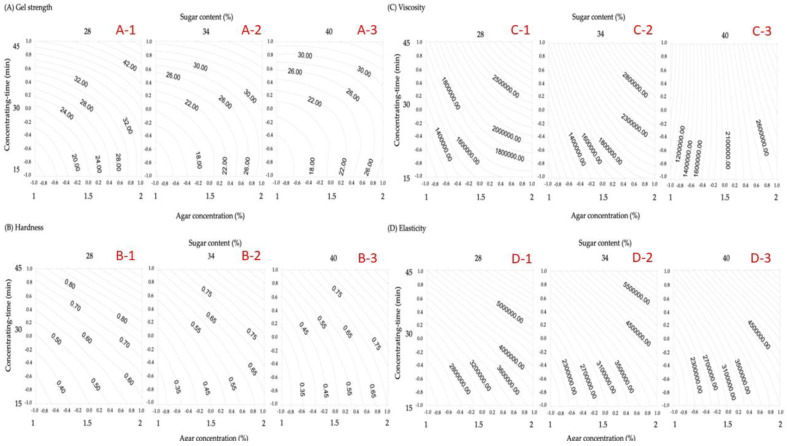
Contour plot of the processing conditions for the gelatinized adzuki-bean cake with respect to gel strength, hardness, viscosity, and elasticity.

**Figure 4:**
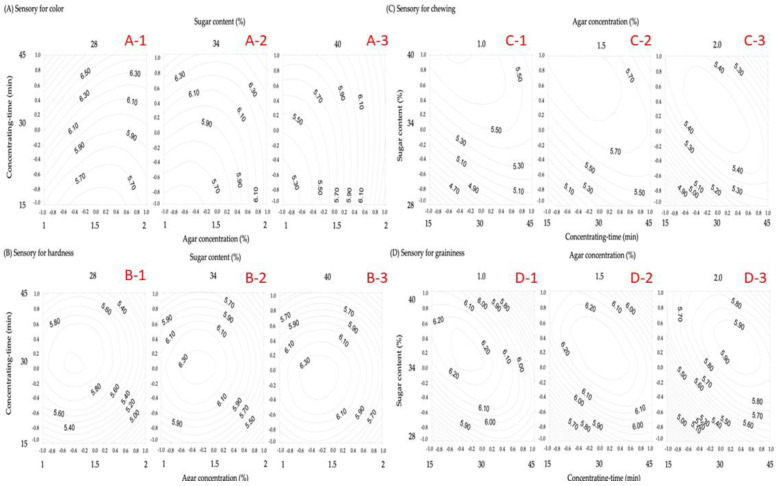
Contour plot of the processing conditions for the gelatinized adzuki-bean cake with respect to sensory evaluation of color, hardness, chewiness, and granularity.

On the basis of polynomials obtained from RSM, the plots of the isotropic curves of each physicochemical property regression model (Figure 2, Figure 3 and Figure 4) were developed using the Surfer software package to investigate and analyze the optimum reaction conditions of the physicochemical properties of gelatinized adzuki-bean cake.

### 3.2. Syneresis (%)

The influences of each processing independent variable on the syneresis (%) of gelatinized adzuki-bean cake were as follows: concentration time > agar concentration > sugar content (Table 5). Concentration time had a significant effect (*p* < 0.05). The syneresis of gelatinized adzuki-bean cake decreased following the extension of concentration time, although a decrease in heat concentration might affect the decrease in syneresis (Figure 2A). Otherwise, the moisture was bound internally with the ingredients or gels, thereby reducing hydration. Gel hysteresis is the loss of moisture from agar hydrogels over time. Aggregation of double helices in agar gels leads to the contraction of the polymer network, reducing the interstitial space available to hold water [18]. Moreover, a study reported that the basic factors affecting syneresis of agar gels were concentration of gel, prolonged storage, apparent gel strength, pressure, and sulfate content [19]. Our team’s previous research showed that adzuki beans contained 10.70% amylose and 0.36% damaged starch [20]. Additionally, previous studies have reported that amylose is insoluble in water and has a less tendency to form gels or pastes, while the water may be released during the reverse grading, leading to syneresis [21]. The phenomenon of syneresis has also been considered undesirable for starch gel products [12].

### 3.3. Chromatic Color

The influence of each process independent variable on the color of gelatinized adzuki-bean cake (Table 5) is as follows: sugar content > agar concentration > concentration time. A significant effect of sugar content was observed (*p* < 0.05). The *L*-value increased with the sugar content at the shorter 15 min concentration time (Figure 2B). At a concentration time of 30–45 min, the *L*-value decreased as the sugar content reduced and agar concentration increased, which is probably caused by the ability of sugar to retain water during a short concentration time, thus increasing the brightness. However, at a long concentration time, the *L*-value decreased because of the caramelization effect, while the agar concentration increased by itself, which increased the *L*-value, thereby increasing the brightness of the gelatinized adzuki-bean cake. The influence of the a-value was ordered as follows: concentration time > sugar content > agar concentration. The a-value of gelatinized adzuki-bean cake decreased as the concentration time increased (Figure 2C). The effect of caramelization on the redness of gelatinized adzuki-bean cake possibly occurred because the water retention effect of sugar prevented water dissipation. Generally, the decrease in the *a*-value of gelatinized adzuki-bean cake was caused by the concentrated time. Notably, the conventional gelatinized adzuki-bean cake is sweetened to 70–72 °brix to reduce water activity by preventing microbial growth [22,23,24]. Furthermore, the Maillard reaction caused by sugar content will influence the color, flavor, sweetness, and texture of the products [25]. This reaction is known as glycation, a nonenzymatic browning reaction occurring from the amino acid residues of proteins and reducing sugars [26]. Depending on the amount of added sugar, the Maillard reaction and caramelization occur through heating, resulting in a darker color and decreased hardness, adherence, and chewiness.

### 3.4. Texture Analysis

The textural properties of food arise from the structural elements of food that are perceived by the sense of touch, related to the deformation, disintegration, and flow of food under forces, and objectively measured by functions of mass, time, and distance [27]. In the texture analysis of gelatinized adzuki-bean cake, each independent processing variable on gelatin strength, hardness, viscosity, and elasticity was investigated (Table 5). The texture was used primarily for solid or semi-solid foods that exhibit hardness and viscosity. Ideal solids exhibit only elasticity (deformation) [27]. The influence on gel strength is concentration time > agar concentration > sugar content, thus indicating the significant effects of the three factors (*p* < 0.001 and *p* < 0.01). The effect on hardness is concentration- time > agar concentration > sugar content. Thus, concentration time and agar concentration had a significant effect (*p* < 0.01). The effect on viscosity is agar concentration > concentration time > sugar content, with agar concentration having a significant effect (*p* < 0.05). The factors affecting elasticity are concentration time > agar concentration > sugar content, with concentration time and agar concentration having a significant effect (*p* < 0.01). The gel strength, hardness, and elasticity of gelatinized adzuki-bean cake showed a positive correlation with the concentration time and agar concentration, which were highly correlated with water dissipation (Figure 3A–D). At 28% and 34% sugar content, the viscosity increases depending on the concentration time and agar concentration, whereas it increased with the concentration time at 40% sugar content. The study’s results are consistent with the observations of Ellis et al. [28]. Interestingly, the sugar concentration variation affected the gelatin-starch gels’ microstructure and aroma release rate [29].

### 3.5. Sensory Evaluation

This study aimed to determine the preferences and overall acceptability of each process-independent variable on the sensory evaluation of color, hardness, chewiness, and granularity. The influence on color preference was as follows: concentration time > agar concentration > sugar content. The color preference increased with the concentration time at lower sugar content (Figure 4A); at high sugar content, the color preference increased with the agar concentration. The influence on hardness preference is concentration time > agar concentration > sugar content. The preference for hardness had the highest score at a 30-min concentration time and agar concentration of approximately 1.2–1.5% (Figure 4B). The effect on chewiness preference is sugar content > concentration time > agar concentration, with sugar content having a significant effect. At low sugar content and short concentration time, the score increased with sugar content and concentration- time (Figure 4C). However, the high sugar content and long concentration time decreased the score due to moisture loss [28]. The results of granularity preferences (Figure 4D) were similar to chewiness preferences. The results of the sensory evaluation preference indicate that the gelatinized adzuki-bean cake produced after 30–40 min of concentrated processing with 34–40% sugar and 1.2–1.5% agar concentration was recognized and appreciated by the experienced evaluators. Generally, commercially available gelatinized adzuki-bean cakes are popular among consumers because of their smooth texture, chewiness, and aroma of beans. However, these cakes are too sweet and high in calories; thus, investigating and improving them is worthwhile. The chewiness of the gelatinized adzuki-bean cake in this study was not similar to that of the commercially available ones. During food formulation, the ingredient modification correlates with the structure [30]. 

### 3.6. Microstructure Observation

**Figure 5:**
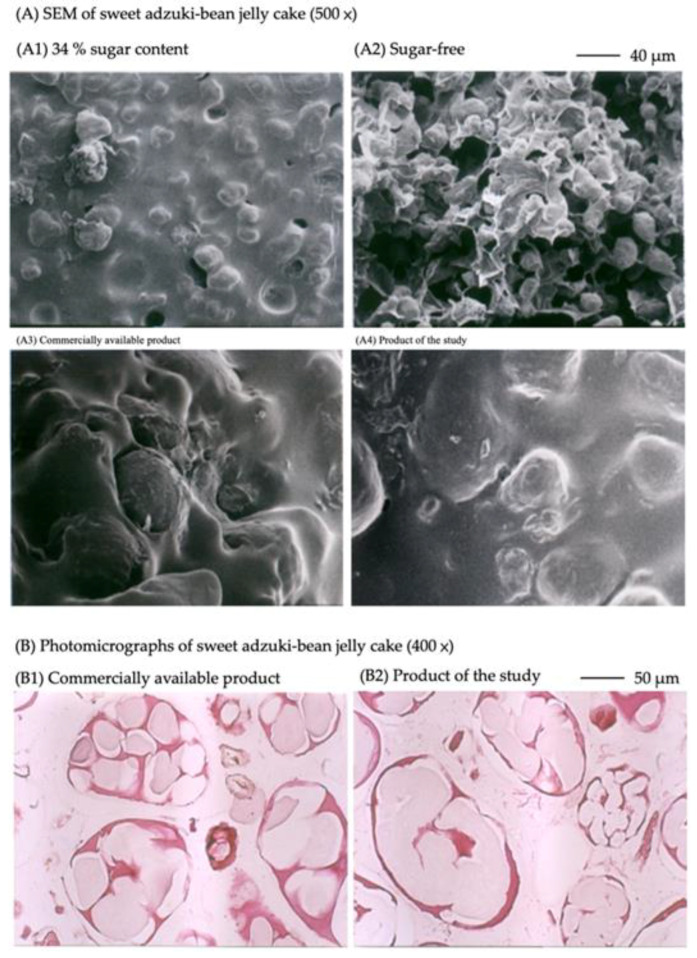
SEM micrographs and photomicrographs of sweet adzuki-bean jelly cake.

Figure 5(A1) shows the SEM micrograph of the gelatinized adzuki-bean cake with 34% sugar, which shows a continuous layer of adhesion on the surface of the adzuki-bean paste granules. Figure 5(A2) shows the microstructure of the gelatinized adzuki-bean cake without added sugar, indicating that only flaky adhesions covered the surface of the adzuki-bean paste granules. Hsieh et al. [31] suggested that the gelatinization of starch in bean paste particles was associated with the texture of bean paste. Furthermore, during the kneading process of bean paste, the cell walls of bean paste would be damaged, thus releasing the starch particles, and a high, stirring force and speed increased the breakdown of bean paste. Gelatinization occurs when starch is heated in the presence of water, which is dispersed into the starch granules and swells due to the loss of crystallinity and molecular sequence caused by the hydration of the amorphous phase [32,33]. Through the dispersion system of foods, the sugar, damaged starch, and agar of gelatinized adzuki-bean cake was dissolved by heating into the continuous phase, which covered the dispersion phase of bean paste particles [20]. Thus, the smoothness and the degree of solubility will create the texture of the gelatinized adzuki-bean cake. Its evenness constitutes the texture of the gelatinized adzuki-bean cake, such as stickiness, elasticity, and chewiness (Figure 5(A1)). However, without sugar, agar plays the role of gelling agent, whereas the moisture in the gel was dried out by the freeze-drying, which only retains the flaky coating, and the gelatinized adzuki-bean cake would lack stickiness, elasticity, and chewiness (Figure 5(A2)).

Bean paste, sugar, and agar are mixed, cooked, and concentrated during processing. Viscosity occurs when more sugar is added, thus influencing the texture and quality. SEM micrographs show that the commercially available gelatinized adzuki-bean cake (Figure 5(A3)) and the product of this study (Figure 5(A4)) exhibit similar microscopic appearance of adhesions covering the bean paste particles. Figure 5(B1,B2) are optical microscope images showing that spaces are found in each bean paste cell, supposedly the adhesions formed by agar and sugar, while starch has broken and dissolved.

### 3.7. Optimal Production Conditions

The above results indicate that agar concentration, sugar content, and concentration time significantly affected the quality of gelatinized adzuki-bean cake, with concentration time having the most significant effect on each property. This finding indicates that the concentration steps, such as the mechanical force of mixing and the concentration endpoint temperature, influenced the quality of gelatinized adzuki-bean cake. The above findings are summarized, and the contour plot of each variable is compiled in Table 5. The optimum processing conditions for gelatinized adzuki-bean cake is an agar concentration of 1.2–1.5%, 34–40% sugar, and 30–40 min concentration time, as indicated by the results of relevant quality property and evaluation preference.

## 4. Conclusions

In this study, the optimal processing conditions for gelatinized adzuki-bean cake were 1.2–1.5% agar concentration, 34–40% sugar content, and 30–40 min concentration time, respectively, as determined by the results of relevant quality properties and evaluation preferences. Hence, this study investigated the optimum processing conditions, quality-related influencing factors, and gelatin properties of gelatinized adzuki-bean cake to provide the required quality-related information to achieve relevant improvements, thus promoting innovative technological advancement in the food industry. The mutual influence between the damaged starch of adzuki-bean pastes with gelatinized adzuki-bean cake and sugar content (or types) during the heating process needs to be investigated in the future.

## Figures and Tables

**Table 1:** Experimental design for three variables—three levels response surface analysis of gelatinized adzuki-bean cake.

X Agar Concentration	Y Sugar Content	Z Concentration-Time	Number of Runs
±1	±1	0	3 × 4 = 12
±1	0	±1	
0	±1	±1	
0	0	0	1 × 3 = 3
Total runs			15

**Table 2:** Process variables and their levels in the three variables—three levels response surface design of gelatinized adzuki-bean cake.

Independent Variables	Coded Symbols	Levels	
		Coded	Uncoded
Agar concentration (%)	X	1	2.0
		0	1.5
		−1	1.0
Sugar content (%)	Y	1	40
		0	34
		−1	28
Concentration-time (min)	Z	1	45
		0	30
		−1	15

**Table 3:** Experimental data for various responses of sweet adzuki-bean jelly cake with different combinations of agar concentration (X), sugar content (Y), and concentration time (Z) used in the design for response surface methodology.

Run ^a^ No	Variable Code Level			Experimental Data for Responses														
	X	Y	Z	SY	LV	AV	BV	GS	HA	VI	EL	SC	SF	SBF	SH	SQD	SG	SOA
1	1	1	0	8.11	11.71	7.93	1.28	28.73	0.8071	2,770,803.00	4,878,917.95	6.63	6.00	6.25	5.50	5.38	5.50	5.75
2	−1	−1	0	13.66	13.35	8.28	1.44	20.28	0.4579	1,485,534.20	2,767,960.02	6.25	6.13	6.50	6.13	4.75	6.13	6.13
3	1	−1	0	5.53	13.63	8.89	1.86	36.10	0.8282	2,622,992.58	5,006,062.15	6.00	5.88	6.00	5.00	5.13	5.13	5.50
4	−1	1	0	20.34	11.73	7.78	0.84	18.70	0.2725	674,228.85	1,752,067.79	5.38	5.88	5.75	6.38	5.63	6.00	5.88
5	1	0	1	5.54	12.95	7.60	0.85	40.16	1.1355	3,902,214.23	6,864,178.18	6.38	6.38	6.25	5.38	5.38	6.13	6.38
6	−1	0	−1	24.17	11.22	8.92	1.88	12.70	0.2605	616,982.80	1,574,723.75	5.75	5.50	5.88	5.25	5.13	5.88	5.25
7	1	0	−1	11.63	12.09	8.10	1.20	29.52	0.5881	1,570,121.70	3,499,844.87	6.25	6.50	6.25	5.13	5.13	5.50	6.00
8	−1	0	1	7.77	9.13	8.11	1.63	34.61	0.6893	1,870,292.03	4,166,362.71	6.63	5.88	6.13	5.25	5.50	5.75	6.00
9	0	1	1	6.46	11.00	7.11	1.19	33.67	0.8448	2,724,622.95	5,106,710.01	6.00	6.00	5.63	5.50	5.38	5.63	5.75
10	0	−1	−1	17.09	13.24	9.29	1.93	21.43	0.4438	1,607,065.74	2,955,168.89	5.50	5.38	6.38	5.13	4.63	5.13	5.00
11	0	1	−1	14.53	10.47	8.31	1.42	19.36	0.5403	1,847,202.00	3,265,685.54	5.50	5.63	5.88	5.88	5.88	6.25	6.00
12	0	−1	1	6.52	12.93	7.88	0.86	44.17	0.8991	2,268,862.90	5,284,201.63	6.75	6.50	6.00	5.50	5.38	5.75	5.88
13	0	0	0	14.52	11.68	8.87	1.85	22.56	0.4954	1,754,841.90	3,309,792.40	5.75	5.63	6.25	6.50	5.50	6.50	6.13
14	0	0	0	17.95	11.21	8.75	2.16	21.90	0.6133	2,301,192.28	3,707,304.00	5.63	5.85	5.88	6.13	5.83	6.22	6.50
15	0	0	0	10.04	10.41	9.05	2.15	24.55	0.6172	2,185,742.08	3,730,830.99	6.25	6.40	5.75	6.38	6.00	6.19	6.25

^a^ The experimental runs were performed in random order.

**Table 4:** Analysis of variance for response variables based on various responses.

Source	DF ^a^	Sum of Square														
		SY	LV	AV	BV	GS	HA	VI	EL	SC	SF	SBF	SH	SQD	SG	SOA
Model	9	419.94	18.98	4.55	2.05	1111.93 ***	0.78 **	8.78 × 10^12^ *	2.68 × 10^13^ **	2.42	0.94	0.65	3.13	1.92 *	1.92	1.64
Linear	3	371.24 *	11.68	3.25 *	0.73	954.05 ***	0.73 ***	8.12 × 10^12^ **	2.54 × 10^13^ ***	1.27 *	0.64	0.28	0.79	0.80 *	0.50	0.50
Quadratic	3	16.36	4.93	1.21	1.14	99.97 **	0.03	1.17 × 10^11^	9.52 × 10^11^	0.31	0.07	0.09	2.17 *	0.62 *	0.83	0.73
Cross product	3	32.34	2.37	0.09	0.18	57.90 *	0.02	5.33 × 10^11^	4.06 × 10^11^	0.84	0.24	0.27	0.17	0.49	0.59	0.42
Residual	5	54.87	3.71	0.81	0.92	9.49	0.03	9.28 × 10^11^	7.83 × 10^11^	0.39	0.84	0.28	0.59	0.16	0.44	0.64
Lack of fit	3	23.40	2.88	0.77	0.86	5.68	0.02	7.62 × 10^11^	6.71 × 10^11^	0.17	0.52	0.14	0.52	0.03	0.38	0.57
Pure error	2	31.47	0.82	0.05	0.06	3.81	0.01	1.66 × 10^11^	1.12 × 10^11^	0.22	0.31	0.13	0.07	0.13	0.06	0.07
Percent of determination (R^2^)		88.44	83.65	84.82	68.97	99.15	96.86	90.43	97.16	86.19	52.97	69.92	84.15	92.48	81.26	71.85

^a^ degree of freedom * Significance at 5%; ** Significance at 1%; *** Significance at 0.1%. SY: syneresis (%), LV: *L*-value, AV: *a*-value, BV: *b*-value, GS: gel strength (g× mm), HA: hardness (g× mm^2^), VI: viscosity (dyn× s× mm^2^), EL: elasticity (dyn× mm^2^), SC: sensory color, SF: sensory fineness, SBF: sensory bean flavor, SH: sensory hardness, SQD: sensory Q degree, SG: sensory graininess, SOA: sensory overall acceptability.

**Table 5:** Analysis of variance for the overall effect of process variables on the various responses.

Source	DF ^a^	Sum of Square										
		SY	LV	AV	GS	HA	VI	EL	SC	SH	SQ	SG
X	4	186.23	6.08	0.49	336.73 ***	0.36 **	5.45 × 10^12^ *	1.28 × 10^13^ **	1.13	1.17	0.42	0.71
Y	4	21.87	12.63	1.81	94.45 **	0.02	2.46 × 10^11^	4.04 × 10^11^	0.84	0.54	1.45 **	1.14
Z	4	246.09 *	2.66	2.52	746.41 ***	0.42 **	3.60 × 10^12^	1.39 × 10^13^ **	1.29	1.81	0.63	0.78

^a^ degree of freedom. SY: syneresis (%), LV: *L*-value, AV: a-value, BV: *b*-value, GS: gel strength (g× mm), HA: hardness (g× mm^2^), VI: viscosity (dyn× s× mm^2^), EL: elasticity (dyn× mm^2^), SC: sensory color, SF: sensory fineness, SBF: sensory bean flavor, SH: sensory hardness, SQD: sensory Q degree, SG: sensory graininess, SOA: sensory overall acceptability. * Significance at 5%; ** Significance at 1%; *** Significant at 0.1%. X: agar concentration (%), Y: sugar content (%), Z: concentration- time (min).

## Data Availability

The authors declare that all data supporting the findings of this study are available within the article.
